# Qualitative Analysis of Polyphenols in Glycerol Plant Extracts Using Untargeted Metabolomics

**DOI:** 10.3390/metabo13040566

**Published:** 2023-04-17

**Authors:** Joseph Robert Nastasi, Venea Dara Daygon, Vassilis Kontogiorgos, Melissa A. Fitzgerald

**Affiliations:** 1 School of Agriculture and Food Sciences, The University of Queensland, Brisbane, QLD 4072, Australia; v.kontogiorgos@uq.edu.au (V.K.); m.fitzgerald2@uq.edu.au (M.A.F.); 2 Queensland Metabolomics and Proteomics Facility, Metabolomics Australia, The University of Queensland, Brisbane, QLD 4072, Australia; v.daygon@uq.edu.au

**Keywords:** glycerol, metabolomics, polyphenols, flavonoids, anthocyanins, chemometrics, UPLC-Q-ToF-MS/MS, MS-DIAL, Queen Garnet Plum, PCA

## Abstract

Glycerol is a reliable solvent for extracting polyphenols from food and waste products. There has been an increase in the application of glycerol over benchmark alcoholic solvents such as ethanol and methanol for natural product generation because of its non-toxic nature and high extraction efficiency. However, plant extracts containing a high glycerol concentration are unsuitable for mass spectrometry-based investigation utilising electrospray ionization, inhibiting the ability to analyse compounds of interest. In this investigation, a solid phase extraction protocol is outlined for removing glycerol from plant extracts containing a high concentration of glycerol and their subsequent analysis of polyphenols using ultra-performance liquid chromatography coupled with quadrupole time of flight tandem mass spectrometry. Using this method, glycerol-based extracts of Queen Garnet Plum (*Prunus salicina*) were investigated and compared to ethanolic extracts. Anthocyanins and flavonoids in high abundance were found in both glycerol and ethanol extracts. The polyphenol metabolome of Queen Garnet Plum was 53% polyphenol glycoside derivatives and 47% polyphenols in their aglycone forms. Furthermore, 56% of the flavonoid derivates were found to be flavonoid glycosides, and 44% were flavonoid aglycones. In addition, two flavonoid glycosides not previously found in Queen Garnet Plum were putatively identified: Quercetin-3-*O*-xyloside and Quercetin-3-*O*-rhamnoside.

## 1. Introduction

Untargeted metabolomics using liquid chromatography-mass spectrometry (LC-MS) is currently the gold standard technique for understanding the complexity of liquid-based crude plant extracts [1,2]. This analytical approach has been enhanced by using mass spectrometer instruments capable of tandem fragmentation [3], often denoted as ‘tandem mass spectrometry’ or ‘MS/MS’. In addition, the utilisation of a time-of-flight tube (ToF) after initial compound ionisation permits the recording of unique fragment parameters correlating fragment size and flight duration or fragment separation by their unique flight time in response to their unique mass [4]. Furthermore, adding a Quadrupole-ToF (Q-ToF) system introduces a collision cell prior to the ToF tube, which can further isolate and fragment specific ions [5]. The combination of both ToF analysis and MS/MS is used to generate comprehensive molecular profiles of complex plant samples where plant metabolites can be identified by their initial mass after ionisation (MS1) and fragment masses (MS2), also commonly denoted as “precursor” and “product ions” respectively [6]. Currently, LCMS-Q-ToF-MS/MS has been considered to have one of the highest coverage for plant metabolite analysis for discovery-type investigations [7]. 

Post-analytical or data processing is one of the biggest challenges of untargeted metabolomic-based investigations due to the large amount of data generated by the procedure [8]. To address this step, large-scale batch annotation procedures utilising machine learning have been developed [9]. However, methods to process the large data sets recorded from untargeted metabolomics constantly evolve [10]. Therefore, annual reviews of current methods and new techniques are needed to improve already established workflows [11]. Currently, there is no standardised workflow for processing the large data sets obtained from high-resolution mass spectrometry-based experiments. However, some steps should be taken to avoid unrealistic data interpretation. In this work, we use the analytical workflows outlined in the publications by Tsugawa, et al. [12], Tsugawa, et al. [13], and Fraisier-Vannier, et al. [14] to process the large number of complex compound features recorded in metabolomic investigations. MS-DIAL, MS-FINDER, and MS-CleanR are relatively new open-access software packages dedicated to mass spectrometry-based feature annotation and deconvolution, and their impact and influence on research design have been well received by the metabolomics community. In this study, we chose this software suite because open-access software removes hurdles often encountered when replicating research using commercial software [15]. 

Currently, there is no routine protocol to remove glycerol from crude plant extracts generated using glycerol as the primary solvent for untargeted metabolomics. Glycerol is an increasingly popular choice amongst chemists as a ‘green’ extraction solvent for increasing natural product yield [16]. Polyphenols are common plant secondary metabolites extracted using glycerol, and they have been the focus of many investigations utilising glycerol-based deep eutectic solvents (DES) [17]. Glycerol is not an ideal sample matrix for mass spectrometry-based investigation utilising electrospray ionization (ESI) because it can induce signal suppression when it is in high concentration [18,19], which can have a negative effect on feature annotation during data analysis. Furthermore, the high viscosity of glycerol plant extracts can alter peak shape and compound separation [20]. One approach to analysing crude glycerol plant extracts is to use centrifugal partition chromatography coupled with nuclear magnetic resonance (NMR) spectroscopy, but this is a costly and specialised procedure and is not readily available in most analytical laboratories [21]. Therefore, a simple solid-phase extraction (SPE) protocol to prepare glycerol samples for LC-MS analysis was investigated, and for this purpose, a polyphenol-rich fruit extract was selected for method development.

Queen Garnet plum (QGP) (*Prunus salicina*) was first developed by Australian plant breeders as a disease-resistant version of the Japanese blood plum [22] and has since received world wild adoption as a ‘superfood’ since its inception in 2001. This superfood status is reinforced by its high antioxidant activity [23], potential health benefits, and polyphenol composition [24]. Overall, the chemical components of QGP may find applications in the food technology and health science industries. For example, the highly pigmented extractions of QGP could be used as a natural colouring agent [25,26] or antioxidant-rich food preservatives [27,28]. As of recent, QGP fruits juices have been used in a range of human-based trials [29,30,31,32] for the proposed beneficial effects that polyphenols have on human health [33]. However, the metabolite profile of QGP has not been extensively investigated, even though anthocyanins and flavonoid derivatives have been reported as the major secondary metabolites responsible for the associated antioxidant activity of its extracts and juices [24,30]. 

In this investigation, polyphenol-rich extracts of QGP generated using mixtures of glycerol and/or ethanol were cleaned using SPE to remove the glycerol and compared using LCMS-Q-ToF-MS/MS via an untargeted metabolomic workflow. It is hypothesised that glycerol performs similarly to ethanol as a liquid solvent for polyphenol extraction and that the major phenolic species present in QGP are retained after the clean-up procedure. The results from this study are presented in new ways to display the polyphenol profile of plant extracts for qualitative-focused metabolomics. The research outcomes will facilitate the exploration of the metabolites present in glycerol plant extractions for natural product research.

## 2. Materials and Methods

### 2.1. Materials

Unless stated otherwise, all organic solvents and Formic Acid (CAS No: 64-18-6) were acquired from ThermoFisher Scientific (Pittsburgh, PA, USA). Cyanidin-3-*O*-glucoside (PhytoLab, Vestenbergsgreuth, Germany, CAS No: 7084-24-4), Rutin (quercetin-3-rutinoside) (PhytoLab, Vestenbergsgreuth, Germany, CAS No: 153-18-4), quercetin (Sigma-Aldrich, St. Louis, MO, USA, CAS No: 117-39-5), and glycerol (Sigma-Aldrich, St. Louis, MO, USA, CAS No: 56-81-5) were sourced from Merck (Rahway, NJ, USA). 

### 2.2. Plant Material and Extraction

QGP extracts were sourced from Native Extracts Pty Ltd. (Alstonville, NSW, Australia). All extractions of QGP were produced using the flesh and skin of raw plums with the seed removed. Extracts (100 mg/100 mL) were created under four solvent conditions: (glycerol 70%/water 30%) (GW), (glycerol 50%/ethanol 25%/water 25%) (GEW), (ethanol 100%) (E), and (ethanol 70%/water 30%) (EW). Glycerol extracts were generated under a pressure-assisted protocol patented as Cellular Extraction^TM^ that utilises pressure gradients and osmotic pressures. A ratio of 1:1 raw material: extraction solvent mixture was used. The crude extracts were hermetically sealed in amber glass vials, transferred to the laboratory, and stored at −80 °C until analysed. 

### 2.3. Glycerol Removal Using Solid Phase Extraction (SPE)

Glycerol was removed from the QGP samples via SPE with hydrophilic-lipophilic balance (HLB) Oasis cartridges (6 cc, 200 mg, 30 µm, SKU: WAT106202). The SPE workflow is as follows: 2 mL of QGP extract was loaded into HLB cartridges with no pre-conditioning and allowed to pass through. Next, a wash step with 1 mL of Milli Q water was loaded into the cartridge and allowed to pass through. This step was repeated to remove all glycerol contaminants from the SPE cartridge. Then, 3 mL of 100% LC-MS grade methanol was used to elute all compounds from the SPE cartridge. The loading, washing, and eluting fractions were collected, evaporated to dryness using an RVC 2-18 CDplus (Christ, Osterode, Germany) set to 40 °C and resuspended in 1 mL of mobile phase 0.1% *v*/*v* formic acid in Milli Q water (Mobile phase A), and filtered with 0.45 μm syringe filters (Phenomenex AF3-3107-52) for metabolite analysis and glycerol quantification.

### 2.4. Glycerol Quantification

Glycerol content was determined using High-Performance Liquid Chromatography coupled with Refractive Index Detection (HPLC-RID). The HPLC system was a Shimadzu Prominence-i LC-2030C 3D equipped with an external RID 20A (Shimadzu, Kyoto, Japan). The column was a Waters Sugar-Pak Column (10 µm, 6.5 mm × 300 mm, SKU: WAT085188) (Milford, MA, USA), and the RID parameters were as follows: analytical, polarity: positive, cell temperature: 40 °C, and response: 1.5 s. Glycerol was eluted at 10.9 min using an isocratic gradient of 100% pure Milli Q water flowing at 0.6 mL min^−1^. The oven temperature was held at 80 °C, and the autosampler was set to 40 °C. The injection volume of the blanks (mobile phase), samples, and standards was 10 µL. Glycerol was quantified using an 8—point calibration curve (R^2^ = 0.9989) (0.05, 0.1, 0.5, 1, 1.5, 2, 2.5, and 3 mg mL^−1^) (*n* = 5). 

### 2.5. HPLC-RID Method Validation

The glycerol quantification method was checked for reliability via interday (*n* = 5) and intraday injections (*n* = 5) of the 8—point calibration curve. The chromatographic data were processed using the Lab Solution software (Shimadzu, Kyoto, Japan). The limit of quantification (LOQ) and limit of detection (LOD) were determined by assessing the standard deviation of the detector responses and the slope for replicate injections of the calibration standards using the following equations: LOD = 3.3 σ/S and LOQ = 10 σ/S, where S = slope and σ = standard deviation.

### 2.6. UPLC-Q-ToF MS/MS Analysis

QGP extracts were analysed using Ultra Performance Liquid Chromatography—Quadropole Time of Flight Tandem Mass Spectrometry (UPLC-Q-ToF-MS/MS). Samples (1 µL) were injected into an ExionLC AC system coupled to a Sciex X500B QToF mass spectrometer (AB SCIEX, Toronto, Canada) with an ESI operating in Data Independent Acquisition (DIA) mode [34]. Analysis was conducted in both positive and negative ionisation modes. Metabolites were separated on a Waters ACQUITY UPLC BEH C18 column (130 Å, 1.7 µm, 2.1 mm × 50 mm, SKU: 186002350) (Milford, MA, USA) and the column oven temperature was held at 45 °C. The mobile phase was 0.1% formic acid in Milli Q water (A) and 0.1% formic acid in 100% methanol (B), flowing at 0.5 mL min^−1^. The mobile phase gradients were: 0–3 min (0% B), 3.0–20.0 min (0–50% B), 20.1–25 min (50–100% B), 25–27 min (100% B), and 27.1–30 (100–0% B). MS source parameters: curtain gas = 35 psi, Gas 1 = 60 psi, Gas 2 = 50 psi, temperature = 500 °C, ion spray voltage 5500 V with DP = 40, and CE = 5 V. ToF-MS/MS parameters were: ToF start = 50 Da, ToF end = 1000 Da, accumulation time = 0.025 s, CE = 35 V ± 15 V. Before analysis QGP samples were cleaned via SPE and filtered using 0.45 μm syringe filters (Phenomenex AF3-3107-52) before injection. Quality Control (QC) samples were made by mixing equal parts of all samples after SPE clean-up. Samples were injected randomly, with QC samples every five injections, and MS instrument calibration was performed every ten injections using the Sciex ESI Positive or Negative Calibration mix and autocalibration function in Sciex OS software.

### 2.7. Processing of LC-MS/MS Data

Data files were extracted from the SCIEX OS Software (AB SCIEX, Toronto, ON, Canada) in the WIFF2 file format and converted into MzML format using MSConvert via the Proteowizard software [35]. Next, the files were uploaded into the portable version of MZmine3.0.21 [36] for chromatogram inspection. This inspection aimed to evaluate the noise threshold level, superimpose QCs and replicate samples for *m/z* drift potential, and analyse any concerning peak information. During this step, minimal variation was observed between replicates, and all samples were considered suitable for analysis. Most importantly, the average minimum peak of the samples was noted for subsequent data processing using MS-DIAL v4.80 [12]. Sample files in the WIFF2 format were imported into MS-DIAL to process the positive and negative ionisation mode datasets. All samples were aligned off a QC file, and the alignment file was normalised by the total ion chromatogram method. The normalisation result, raw peak height data, and detected feature information were extracted for feature filtering using the MS-CleanR software package [14]. After feature filtering, the peak results were exported into MS-FINDER [13] for compound annotation via formula prediction and structure elucidation by an in silico fragmenter cross-referenced with the following spectral databases: FoodDB [37], ChEBI [38], NANPDB [39], KNApSAcK [40], COCONUT [41], and PubChem [42]. After compound annotation, a single file containing the top compound candidates for each peak up to a maximum of ten compounds was exported from MS-FINDER and into MS-CleanR to merge the positive and negative ionisation mode results following recommendations from Fraisier-Vannier, Chervin, Cabanac, Puech, Fournier, Durand, Amiel, André, Benamar and Dumas [14]. During this step, annotations assigned for each peak and cluster were ranked based on their MS-FINDER score and not on a database ranking. After compound and cluster merging, a final peak list of all compounds with other possible annotations was exported and manually curated. During this step, potential annotations were selected based on the MS-Finder score, reasonability to be the annotated compound, and cross-referencing with the MS2 spectra in the MS-Dial software. Compounds fragmented in positive and negative ionisation modes were resolved based on their highest abundance across the sample groups. However, if a compound had a higher peak abundance in 70% of the individual injections in either positive or negative ionisation mode, that ionisation mode was selected for that peak. To further screen for polyphenols, the ontology of the annotated features was assessed, and only polyphenol derivatives were included for data analysis.

### 2.8. Statistical Analysis

Chemometric analysis of the QGP metabolite data was performed using SIMCA 17 (Umetrics, Sweden). Principal Component Analysis (PCA) models were generated using the putatively annotated peak list exported from the MS-CleanR software package. Cross Validation (CV) for model calibration was performed using SIMCA 17 standard protocol (G = 7, leave one out). The left-out group is predicted using Predictive Residual Sum of Squares (PRESS) and repeated G times to determine the overall PRESS value of the model. PCs generated for the PCA were considered significant if they met the criteria for Rule (R1) as determined by SIMCA 17 protocols. Using the autofit option for model calibration, the appropriate number of PCs was chosen for the deconvolution of the data sets. The ‘Q^2^’ value is determined by the function Q^2^ = 1 PRESS/SS where PRESS = Σ (observed − predicted)^2^ and SS is the sum of squared deviations from the mean of X. Q^2^ can be used to support the predictiveness of a component and Q^2^ close to that of R^2^ indicates good predictability. 

## 3. Results

### 3.1. HPLC-RID Glycerol Quantification

**Figure 1:**
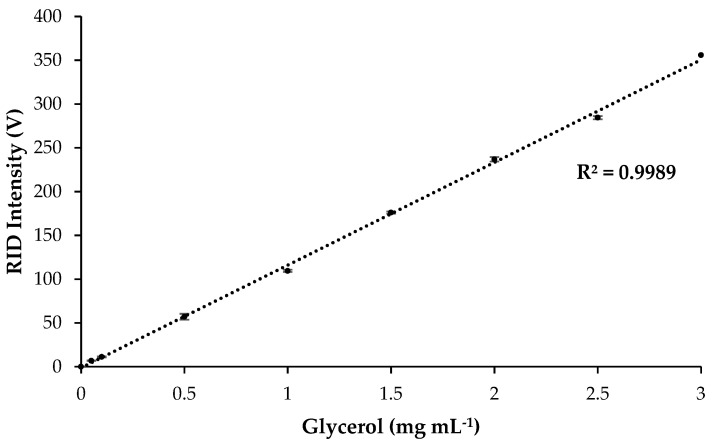
Calibration curve for glycerol quantification. Standards injections were tested for interday (*n* = 5) and intraday (*n* = 5) reliability. Error bars represent standard deviation across interday and intraday injection for each point in the calibration curve (*n* = 25). LOD = 0.13 mg mL^−1^, LOQ = 0.39 mg mL^−1^.

Table 1 reports the glycerol content of QGP extracts (GW and GEW) after SPE clean-up. The EW and E extracts did not contain glycerol and therefore were not cleaned using SPE. SPE clean-up of the GW and GEW extracts had a similar pattern of glycerol removal, where the load fractions contained most of the glycerol, and trace amounts were present in the successive washing steps using water. The final elution step with methanol contained <1 mg mL^−1^ of glycerol for both the GW and GEW extract. After SPE clean-up, there was <1% *w*/*v* glycerol remaining in the QGP extracts, which are expected to not interfere with the ESI source of the mass spectrometer. The recovery percentage of glycerol in the GW and GEW extracts was 99.7% and 99.1%, respectively. The calibration curve (R^2^ = 0.99) used for quantifying glycerol in the QGP samples is displayed in Figure 1 (LOD = 0.13 mg mL^−1^, LOQ = 0.39 mg mL^−1^). Interday (*n* = 5) and intraday (*n* = 5) injections of the glycerol standards had low standard deviations for each point in the calibration curve.

### 3.2. Untargeted Metabolic Profiling of QGP Extracts Using UPLC-Q-ToF-MS-MS

**Figure 2:**
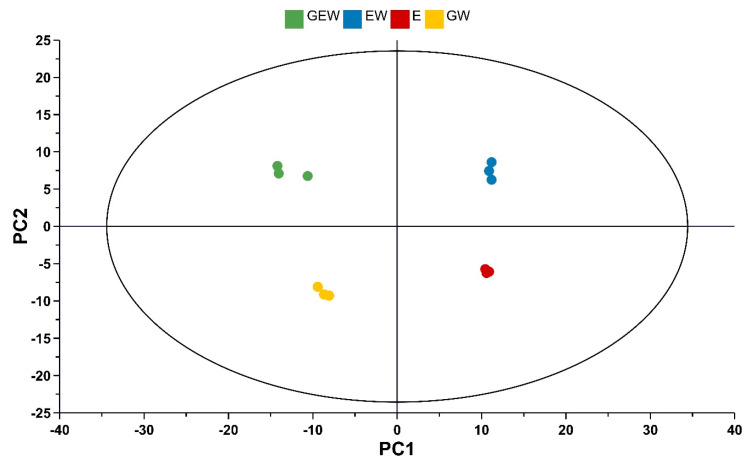
PCA scores plot of QGP extracts. E = ethanol, EW = ethanol/water, GEW = glycerol/ethanol/water, and GW = glycerol/water. Extracts are coloured by their respective groups (*n* = 3) and sized by their DCrit value. DCrit is a unitless value that represents a limit for potential outlier identification. Identical sizing of all circles indicates no potential model outliers and standard variation between replicates. Four significant PCs were generated for this model where the following variance has been explained; PC1: 46.1%, PC2: 21.6%, PC3: 13.4%, PC4: 8.0%, and R^2^ cumulative: 89%. QGP extracts generated using glycerol as a solvent separate along PC1 from ethanolic extracts. GW and EW separate from GW and E along PC2. GW separates along PC1 and PC2 from EW indicating significant group differences. Separate grouping of extraction solvents into the centre of each quadrant demonstrates ‘like’ variance between groups and even distribution within the model space. Q^2^ cumulative = 66.7% indicates good predictability of the PCA model.

**Figure 3:**
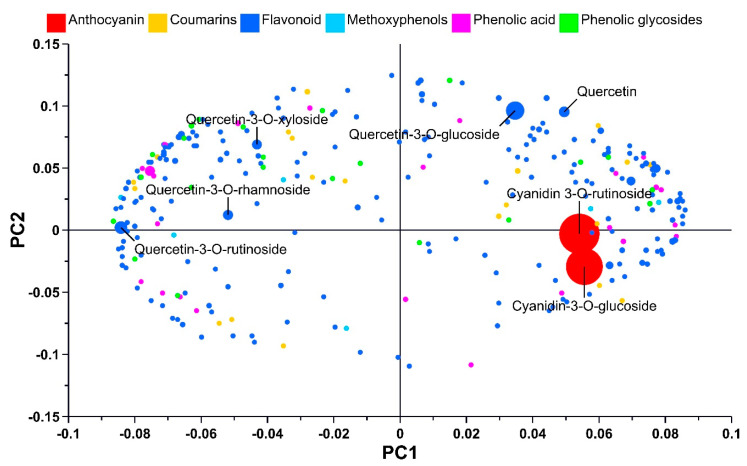
PCA loadings plot from QGP extracts. Metabolites are coloured by their class and scaled for size by the relative abundance of a QC sample. Metabolites in high abundance appear as larger dots than metabolites in low abundance. Cyanidin-3-*O*-glucoside and Cyanidin-3-*O*-rutinoside are very high in abundance compared to the other polyphenols. Flavonoids are the most common polyphenol present in QGP ethanolic and glycerol extracts. Phenolic acids, phenolic glycosides, methoxy phenols, and coumarins are less abundant than flavonoid derivatives.

The phenolic compounds in QGP extracts generated using mixtures of glycerol, ethanol, and water were analysed by UPLC-Q-ToF-MS-MS, and 670 shared peaks were putatively identified across the extracts. The samples were screened and annotated using the MS-Dial, MS-Finder, and MS-CleanR software suites [12,13,14]. A total of 284 compounds were putatively identified as polyphenols via in silico fragmentation in the MS-Finder software and MS2 matching using the databases aforementioned in Section 2.7. Unsupervised analysis via PCA was used to compare the metabolite profiles of triplicate samples of the QGP extracts groups (Figure 2). The PCA scores plot demonstrates clear and distinct clustering of the extracts based on their solvent extraction. The PCA model explained 89% of the variance in the metabolomic data set across four PCs. PC1 explains 46.1% of the variance, and QGP extracts generated using glycerol separate from ethanolic extracts along PC1. PC2 explains 21.6% of the variance, and GEW and EW extracts separate from GW and E along PC2. PC3 and PC4 do not provide any extra information that enhances model interpretation. The separation of the QGP extracts based on their extraction solvents indicates differences in the abundance of their polyphenols but no differences in the presence of specific polyphenols. The PCA was further analysed in the loadings plot (Figure 3) to observe the differences in polyphenol abundance.

The metabolites in the loadings plots are coloured according to the major polyphenol classes and scaled for size to indicate their relative abundance. The loadings show a wide diversity of flavonoids, coumarins, phenolic acids, phenolic glycosides, and methoxy phenols in the QGP extracts. Flavonoids (blue symbols) are the most abundant polyphenol species in the QGP extracts, and two anthocyanins (red symbols)–cyanidin-3-*O*-glucoside and cyanidin-3-*O*-rutinoside–are in very high abundance. Other flavonoids glycosides such as quercetin-3-*O*-rutinoside, quercetin-3-*O*-glucoside, quercetin-3-*O*-xyloside, quercetin-3-*O*-rhamnoside, and one flavonoid aglycone–quercetin–were also in high abundance in the QGP extracts. The compound information for these anthocyanins and flavonoids is shown in detail in Table 2 according to the best reporting practices [43,44]. 

### 3.3. Characterisation and Comparison of Abundant Polyphenols

**Figure 4:**
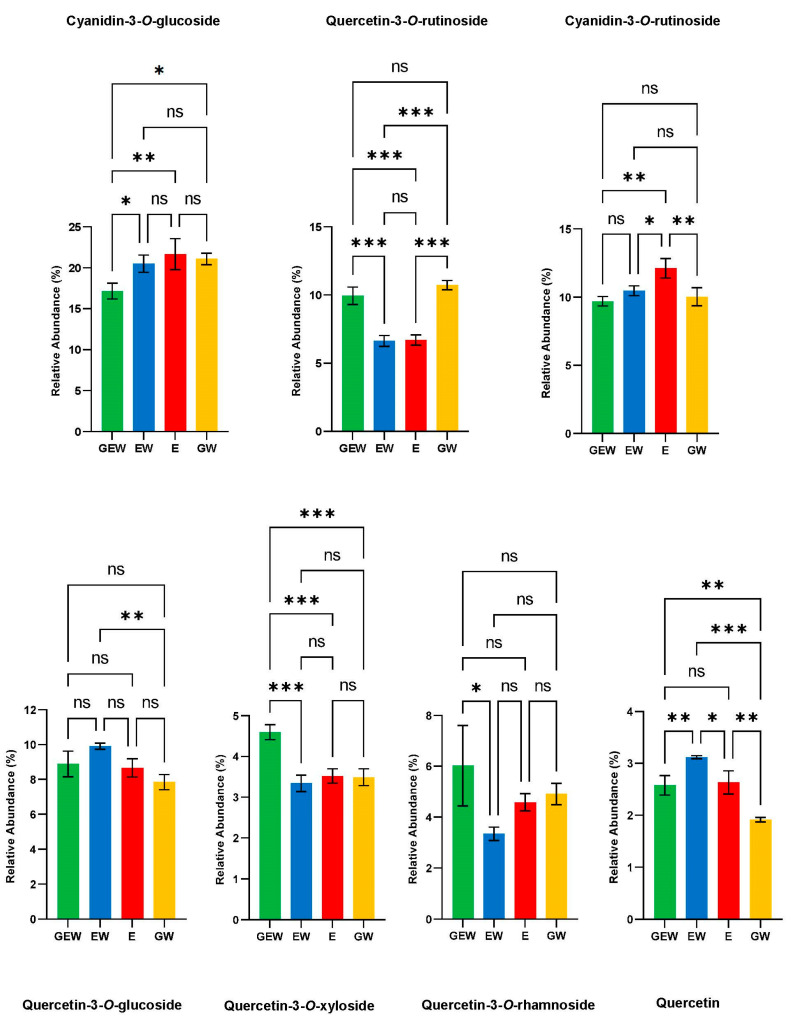
Relative abundance of major polyphenols in Queen Garnet Plum extracts. E = ethanol, EW = ethanol/water, GEW = glycerol/ethanol/water, and GW = glycerol/water. Data are expressed as relative abundance peak area (%) of a metabolite relative to the total peak area of all detected polyphenols (*n* = 284). Samples are expressed as the average of replicates (*n* = 3) and standard deviation is expressed as error bars on the bar graph. Post-hoc analysis between individual groups using Tukey (*p* = 0.05) is displayed above each bar graph. *p* value style: >0.12 (ns), <0.033 (*), <0.002 (**), and <0.001 (***).

Cyanidin-3-*O*-glucoside, quercetin-3-*O*-rutinoside, and quercetin were identified using analytical standards, and the other compounds in Table 2 were putatively identified using their fragmentation patterns. The flavonoid glycosides exhibited characteristic loss of their sugar moieties, which is reported in the MS/MS ES (+)/(−) fragments column of Table 2. Quercetin-3-*O*-rutinoside yielded two fragment ions of 465.101 *m/z* and 303.054 *m/z* which corresponds to the successive removal of the rhamnoside [M  +  H^+^ − 146] and glucoside [M  +  H^+^ − 162] moieties from the precursor ion (611.155 *m/z*) in positive mode. Quercetin-3-*O*-glucoside produced a fragment ion in positive mode at 303.052 *m/z,* corresponding to a quercetin aglycone after removing glucoside moiety [M + H^+^ − 162] from the precursor ion. Quercetin-3-*O*-xyloside yielded a fragment ion at 301.019 *m/z,* which matches the loss of xyloside moiety [M − H^+^ − 132] from the precursor ion (433.075 *m/z*) in negative mode. In addition, the Quercetin-3-*O*-rhamnoside precursor ion (449.104 *m/z*) produced a fragment 303.052 *m/z* characteristic of the loss of a rhamnoside [M  +  H^+^ − 146] moiety. This was distinctly different from the fragmentation pattern of cyanidin-3-*O*-glucoside, which produced a fragment ion of 287.051 *m/z*, reflecting the loss of glucoside moiety [M +  − 162] from the 449.101 m/z precursor ion in positive mode. The cyanidin-3-*O*-rutinoside precursor ion in positive mode (595.161 *m/z*) yielded fragments at 449.109 *m/z* and 287.054 *m/z,* which are reflective of the loss of a rhamnoside [M  +  − 146] and glucoside [M +  − 162] moiety respectively. Quercetin produced multiple fragment ions, which reflected the following losses from the precursor ion (303.047 *m/z*) in positive mode; 257.044 *m/z* [M  +  H^+^ − C_2_H_2_O_2_], 229.049 *m/z* [M + H^+^-CO], 201.054 *m/z* [M + H^+^-CO], and 153.018 *m/z* [M + H^+^-C_5_H_4_]. The relative abundance of each of these polyphenols in the different extraction solvents is shown in Figure 4. 

The different extracts of QGP had consistent relative abundances of the major polyphenols (Figure 4). The relative abundances were determined by comparing the peak area of each compound with the total peak area of all the polyphenols detected in the QGP extracts (*n* = 284). The compounds, in order of their relative abundance from largest to smallest, were cyanidin-3-*O*-glucoside > cyanidin-3-*O*-rutinoside > quercetin-3-*O*-rutinoside > quercetin-3-*O*-glucoside > quercetin-3-*O*-rhamnoside > quercetin-3-*O*-xyloside > quercetin, and this order was maintained across all four extraction solvents. Notably, ethanol extracts of QGP contained the highest percentage of anthocyanins. In comparison, glycerol extracts had higher amounts of quercetin-3-*O*-rutinoside, quercetin-3-*O*-rhamnoside, and quercetin-3-*O*-xyloside, but this was not the same for other quercetin glycoside derivatives. Quercetin was present in a lower abundance compared to its glycoside derivatives. Overall, the information in Figure 4 demonstrates that ethanol, glycerol, or combinations of both solvents all produce extracts with similar abundances of the major polyphenols present in QGP. Other polyphenol species present in QGP are reported in the next section.

### 3.4. Qualitative Analysis of Polyphenols in Queen Garnet Plum

**Figure 5:**
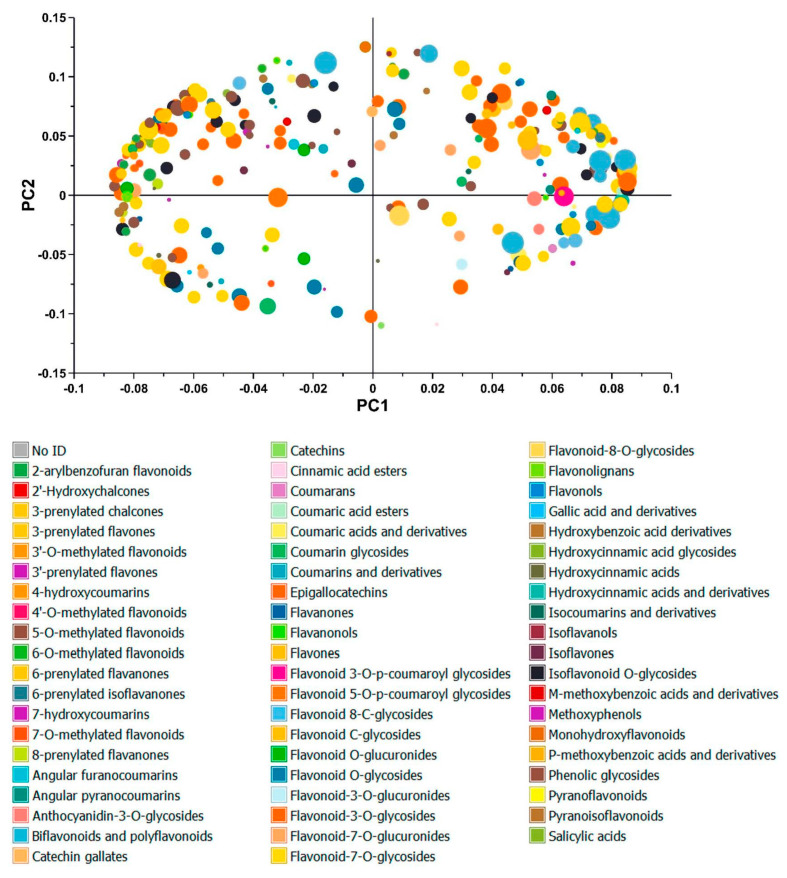
PCA loadings plot of QGP extracts coloured by polyphenol species and size. Polyphenols are coloured by their ontology and scaled by their molecular weight. Major ontologies include flavonoids, flavones, chalcones, isoflavones, flavanones, flavanonols, flavonols, isoflavanols, catechins, coumarins, hydroxybenzoic acids, hydroxycinnamic acids, and various flavonoid glycoside configurations which are attached at different locations on the flavonoid skeleton structure (e.g., Flavonoid-3-*O*-glycosides and Flavonoid-7-*O*-glycosides). The smallest and largest phenolic compounds were 138 *m/z* (Salicylic acid) and 898 *m/z* [Gallocatechin(4alpha->8)]2catechin respectively.

**Figure 6:**
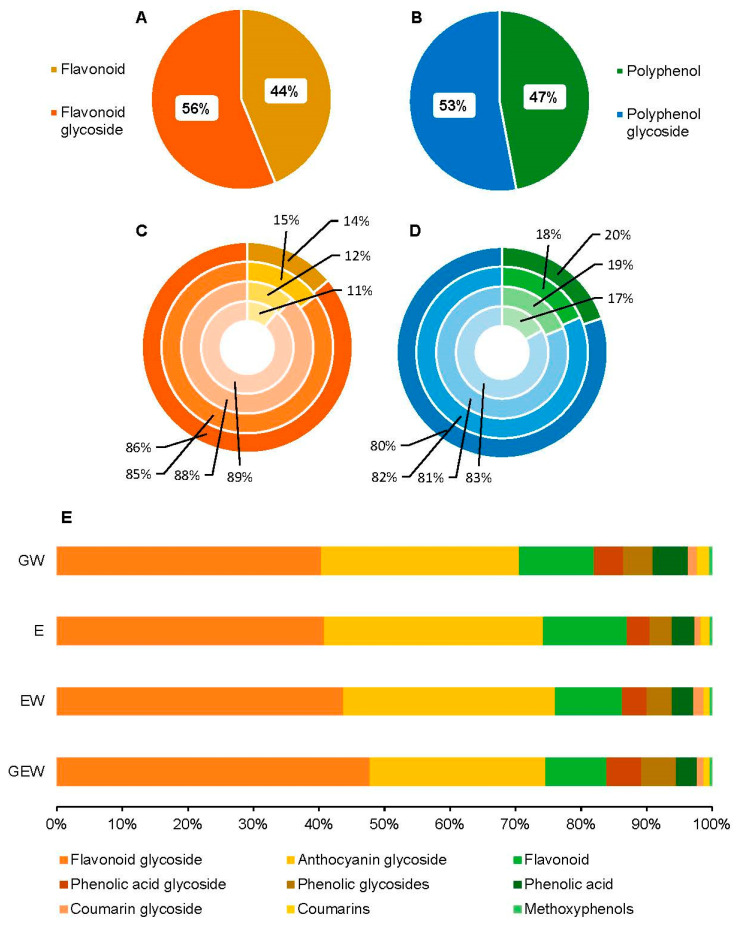
(**A**–**E**) Ontology breakdown of aglycone and glycosidic forms of the polyphenols (*n* = 284) in QGP extracts. Top pie charts show the ratio of flavonoid: flavonoid glycoside (**A**) and polyphenol: polyphenol glycosides (**B**) in QGP. Stacked pie charts (middle) shown the relative abundance of flavonoid (**C**) and polyphenol aglycones and glycosides (**D**) across the four extraction solvents. The extractions solvents are in order from inner to outer circle; GEW, EW, E, GW. The breakdown of the nine major phenolic classes found in QGP are shown in the horizontal bar graph (bottom) (**E**).

Qualitative untargeted metabolomic analysis of QGP yielded 61 unique polyphenol ontologies (Figure 5). In Figure 5, the loadings plot from Figure 3 has been colour scaled for compound ontology and compound size (*m/z*) to enable easier visualisation of the polyphenol species. Most of the polyphenols in QGP were identified as flavonoid-3-*O*-glycosides, flavonoid-7-*O*-glycosides, and flavones. Furthermore, the polyphenol diversity of QGP is dominated by larger molecular weight polyphenols > 400 *m/z*. In Figure 6, the breakdown of phenolic species is further examined by evaluating the ratios of their aglycone and glycone forms. Approximately 56% of the flavonoid derivates in QGP are flavonoid glycosides, and 44% are flavonoid aglycones (Figure 6a). In addition, 47% of all polyphenols in QGP existed in their aglycone form, whereas 53% were glycoside derivatives (Figure 6b). The relative abundance of flavonoid and flavonoid glycosides in the QGP extracts was 11–15% and 85–89%, respectively (Figure 6c). The relative abundance of polyphenols and polyphenol glycosides was 17–20% and 80–83%, respectively (Figure 6d). Therefore, in QGP, approximately 50% of all polyphenols, specifically flavonoids, existed in their glycoside form, where the relative abundance of all flavonoids and all polyphenol derivates was > 80%. The horizontal bar graph shows the relative abundance of the nine major polyphenol classes detected in QGP (Figure 6e), which was consistent across all extraction solvents.

## 4. Discussion

### 4.1. Glycerol Removal for UPLC-Q-ToF-MS/MS Analysis

In the present study, mixtures of glycerol, water, and ethanol were used to extract the polyphenols from the skin and flesh of raw QGPs for untargeted metabolomic investigation. The GEW and GW extracts contained 74.4% and 52.5% glycerol (Table 1) in their extracts, respectively, which is too concentrated for UPLC-Q-ToF-MS/MS analysis without dilution; however, diluting the extracts will reduce the compound resolution. Therefore, an SPE protocol for removing glycerol was investigated using HLB Oasis cartridges to permit the investigation of the secondary metabolite profile of QGP extracts containing glycerol. HLB cartridges work on the principle of hydrophilic-lipophilic balance and utilise N-vinylpyrrolidone and divinylbenzene derivatives as their hydrophilic and lipophilic polymers, respectively [45]. The polymer was capable of absorbing polyphenols which have less relative polarity than glycerol (0.812) and water (1.000) [46]. The polyphenols were then desorbed from the polymer by washing with a solvent of lesser polarity (methanol, 0.762). Table 1 describes the process of the SPE clean-up method to remove glycerol and retain the polyphenols on the HLB cartridge. After a successive two-step washing procedure with water, the glycerol was removed from the cartridge, and the final elution step of the polyphenols contained < 1 mg mL^−1^ of glycerol. Most of the glycerol passed through the HLB cartridge during the loading step, but successive washes with water were required to remove trace amounts bound to the cartridge.

To the best of our knowledge, the application of HLB cartridges to remove glycerol from plant extracts containing >50% glycerol and the subsequent analysis of the extract using high-resolution mass spectrometry has not been previously explored. Therefore, applying this SPE clean-up method enables new opportunities to analyse polyphenols acquired from glycerol and glycerol-based DES mixtures [17]. Previously, the removal of glycerol to allow for compound analysis has been identified as a shortcoming in adopting glycerol over benchmark alcoholic solvents [17]. Application of glycerol as a primary or co-solvent for the extraction of polyphenols from fruits and their waste has been conducted on grapefruit peels [47], bottle gourd fruit [48], chinaberry [49,50], black chokeberry [51], eggplant peel [52], orange peel [53], and red grape pomace [54]. However, none of these studies reports a method to remove glycerol during sample preparation or utilises UPLC-Q-TOF-MS-MS analysis. In a singular study, [55], UPLC-Q-TOF-MS-MS analysis was employed to analyse glycerol-based lemon peel extracts to identify polyphenols. However, the glycerol content of these extracts did not reach the concentrations of the QGP extracts used in the present study. Therefore, for the future analysis of fruit extracts generated using glycerol and glycerol-based DES mixtures, the present SPE clean-up and metabolomics workflow can provide further insight into profiling of the secondary metabolites and provide higher resolution for compound analysis. In the next section, the application of untargeted metabolomics for the qualitative investigation of polyphenol species present in QGP is discussed.

### 4.2. Untargeted Metabolomics of Queen Garnet Plum Polyphenols

Untargeted metabolomics was used to compare the polyphenol profile of the QGP extracts under two premises. Firstly, to evaluate the SPE protocol efficacy in retaining the major polyphenols present in QGP and, secondly, to investigate the polyphenol metabolome of QGP. Using UPLC-Q-ToF-MS-MS analysis and chemometric evaluation, QGP extracts revealed no major differences in their polyphenol diversity but differences in the relative abundance of the polyphenols. This is consistent with the literature investigating glycerol as a co-solvent for polyphenol extraction [17,54]. Therefore, regardless of the extraction solvent used (GEW, EW, E, or GW), the polyphenols species extracted from QGP were consistent. Furthermore, the SPE clean-up method was validated by this analysis, and the polyphenols present in ethanol extracts were also present in the glycerol extracts post-SPE clean-up. The loadings plot in Figure 3 highlights the major polyphenols present in QGP, and the compound fragmentation information of the highlighted compounds is presented in Table 2. The differences in the relative abundance of the major polyphenols in QGP are shown in Figure 4, and the diversity of polyphenols present in QGP is graphically shown in Figure 5 and Figure 6.

Two anthocyanidin-3-*O*-glycosides (cyanidin-3-*O*-glucoside and cyanidin-3-*O*-rutinoside), four flavonoid-3-*O*-glycosides (quercetin-3-*O*-rutinoside, quercetin-3-*O*-glucoside, quercetin-3-*O*-xyloside, quercetin-3-*O*-rhamnoside), and one flavonol (quercetin) were identified as the major polyphenols in the QGP. Collectively, these metabolites comprised over 50% of the relative abundance of polyphenols present in QGP (Figure 4). Previously, QGP has been reported to contain 180.7 ± 23 mg/100 g of anthocyanins (a combination of cyanidin-3-*O*-glucoside and cyanidin-3-*O*-rutinoside) and 56.1 ± 8.3 mg/100 g of quercetin glycosides (combination of quercetin-3-*O*-rutinoside, quercetin-3-*O*-glucoside, and quercetin-3-*O*-galactoside) [24]. A high abundance of anthocyanins compared to quercetin glycosides is consistent with our findings. However, quercetin-3-*O*-xyloside and quercetin-3-*O*-rhamnoside were found in higher abundance than quercetin-3-*O*-galactoside which was detected at 0.25% relative abundance. Previously, quercetin-3-*O*-xyloside and quercetin-3-*O*-rhamnoside have not been reported in the literature to be present in QGP, however, they were found in significant abundance in our investigation. Qualitative inspection of the polyphenol species in QGP revealed 61 unique polyphenol classes, most of which were dominated by flavonoid derivatives (Figure 5). Specifically, QGP is comprised of primarily flavonoids, and specifically, flavonoid glycosides, which were found to be > 80% of the total polyphenol abundance across all four extraction solvents (Figure 6e). QGP has been recently investigated for its potential to reduce metabolic syndrome [30,31,32,56] and the results from the present study can provide insight into these applications. For example, the results from Figure 4, Figure 5 and Figure 6 highlight the diversity, abundance, and ratio of flavonoid aglycones and glycosides in QGP. Flavonoid aglycones have been determined to have higher immune-regulatory activity in vivo because they have greater cellular absorption via passive absorption in the intestinal epithelium [57]. Therefore, while QGP is considered a ‘super food’ because of a significantly high flavonoid content in comparison to other plums [22], the diversity of its polyphenol profile suggests that direct ingestion may result in a lower bioavailability compared to other fruits rich in flavonoid aglycones [58].

### 4.3. Significance of the Results and Future Applications of Glycerol Plant Extracts

Untargeted metabolomic investigations usually follow a typical research paradigm where a treatment effect is investigated across several groups. The up or down-regulated metabolites are investigated, resolved with analytical standards, and finally quantified. Here, we propose a workflow for qualitatively evaluating polyphenols acquired using glycerol-based DES extraction from a flavonoid-rich fruit. Furthermore, this workflow can be extended to all types of plant extracts from fruits, flowers, leaves, and roots. Glycerol is a viable alternative to other benchmark solvents, such as ethanol and methanol, for the generation of plant extracts with wide application in the medical, food, cosmetic, and agriculture industries [49,59]. Advancements in the analytical methods used to analyse glycerol plant extracts aid in establishing a circular economy by reducing waste and promoting eco-friendly solvents [60]. 

Identifying metabolites in glycerol plant extracts will also aid in furthering their application in the food packaging industry, where biopolymer films are now routinely generated using glycerol as a plasticising agent [28]. Incorporating glycerol plant extracts containing antioxidant or antimicrobial-rich extracts into biopolymer film formulations can have a dual effect as plasticising and food-preserving agents. Currently, this is not a widely explored application of glycerol plant extracts, but future research will see the application of glycerol plant extracts as potential novel plasticising agents for biopolymer film development. 

## 5. Conclusions

We have successfully developed an SPE-UPLC-Q-ToF-MS/MS method to analyse polyphenols in glycerol plant extracts. This was validated by comparing the major polyphenols in four QGP extracts generated using mixtures of glycerol, ethanol, and water and removing more than 99% of the glycerol from the plant extracts. After SPE clean-up of the glycerol, the glycerol-containing extracts had no differences in the diversity of their polyphenols compared to ethanol extracts but small variations in their relative abundances. In addition, we identified two new flavonoids in QGP, quercetin-3-*O*-xyloside and quercetin-3-*O*-rhamnoside, which have not been reported in the literature. Furthermore, the polyphenol profile of QGP was determined to be comprised of flavonoids, specifically flavonoid-3-*O*-glycosides and flavonoid-7-*O*-glycosides derivatives. Overall, the polyphenol metabolome of QGP is 53% polyphenol glycoside derivatives and 47% polyphenols in their aglycone forms.

## Figures and Tables

**Table 1:** Glycerol content of SPE fractions from QGP extracts (GW and GEW).

Step	SPE Fractions	Volume (mL)	Glycerol Content (mg mL^−1^)	
			GW	GEW
1	Load	2	705.4 ± 6.82	492.65 ± 9.95
2	Wash H_2_O	1	33.63 ± 5.2	22.77 ± 10.75
3	Wash H_2_O	1	2.73 ± 0.89	2.16 ± 2.02
4	Elute MeOH	3	0.52 ± 0.11	0.48 ± 0.05
			742.29 ± 8.3	520.73 ± 5.42
Glycerol content before SPE (mg mL^−1^):			744.21 ± 1.1	525.25 ± 0.9
Recovery (%):			99.7%	99.1%

GW = glycerol/water and GEW = glycerol/ethanol/water. H_2_O = Water, MeOH = methanol. Values are expressed as average ± standard deviation (*n* = 3).

**Table 2:** Abundant anthocyanins and flavonoids present in Queen Garnet Plum extracts.

RT (min)	Adduct	Putative Metabolite Name	Metabolite Class	Molecular Formula	ES Theoretical *m/z*	ES Found *m/z*	*m/z* Error (Da)	MS/MS ES (+)/(−) Fragments	References (ID)	ID (1–4)
8.85	[M+] ^+^	Cyanidin-3-*O*-glucoside	Anthocyanidin-3-*O*- glycosides	C_21_H_21_O_11_	449.101	449.106	0.002	449.106 (M+), 287.051 (M+ − Glu)	standard	1
9.53	[M+] ^+^	Cyanidin-3-*O*-rutinoside	Anthocyanidin-3-*O*- glycosides	C_27_H_31_O_15_	595.161	595.163	−0.017	595.161 (M+), 449.109 (M + − Rha), 287.054 (M + − Rha − Glu)	441674	2
13.13	[M + H] ^+^	Quercetin-3-*O*-rutinoside	Flavonoid-3-*O*- glycosides	C_27_H_30_O_16_	611.155	610.153	−0.017	611.155 (M + H), 465.101 (M + H − Rha), 303.054 (M + H − Rha − Glu)	standard	1
13.18	[M + H] ^+^	Quercetin-3-*O*-glucoside	Flavonoid-3-*O*- glycosides	C_21_H_20_O_12_	465.097	464.095	0.006	465.097 (M + H), 303.052 (M + H − Glu)	5280804	2
14.06	[M − H] ^−^	Quercetin-3-*O*-xyloside	Flavonoid-3-*O*- glycosides	C_20_H_18_O_11_	433.075	434.085	0.003	433.075 (M − H), 301.019 (M − H − Xyl)	5321278	2
13.27	[M + H] ^+^	Quercetin-3-*O*-rhamnoside	Flavonoid-3-*O*- glycosides	C_27_H_30_O_16_	449.104	448.101	0.004	449.104 (M + H), 303.052 (M + H − Rha)	5280459	2
13.2	[M + H] ^+^	Quercetin	Flavonol	C_15_H_10_O_7_	303.047	302.043	0.003	303.047 (M + H) 257.044 (M + H − C_2_H_2_O_2_), 229.049 (M + H − CO), 201.054 (M + H − CO), 153.018 (M + H − C_5_H_4_)	standard	1

CE (eV) = 35, Glu = Glucose, Rha = Rhamnose, Xyl = Xylose, Reference ID = PubChem database, ID (1–4): 1 = analytical reference standard, 2 = putative identification using library/database/literature, 3 = tentative identification using MS1 spectra, and 4 = unknown [44].

## Data Availability

The data presented in this study are available on request from the corresponding author. Data is not publicly available due to privacy.
